# Supplementary material for: Association between oral microbiome and seven types of cancers in East Asian population: a two-sample Mendelian randomization analysis
Source: Front Mol Biosci. 2023 Nov 21;10:1327893. doi: 10.3389/fmolb.2023.1327893 (PMC10702768; doi:10.3389/fmolb.2023.1327893)
Supplement: Supplementary file 1 [file DataSheet1.ZIP › Supplementary Materials/MR plots of saliva/saliva═╝/Breast cancer/pheno.727_to_breast cancer_leave_one_out.pdf]

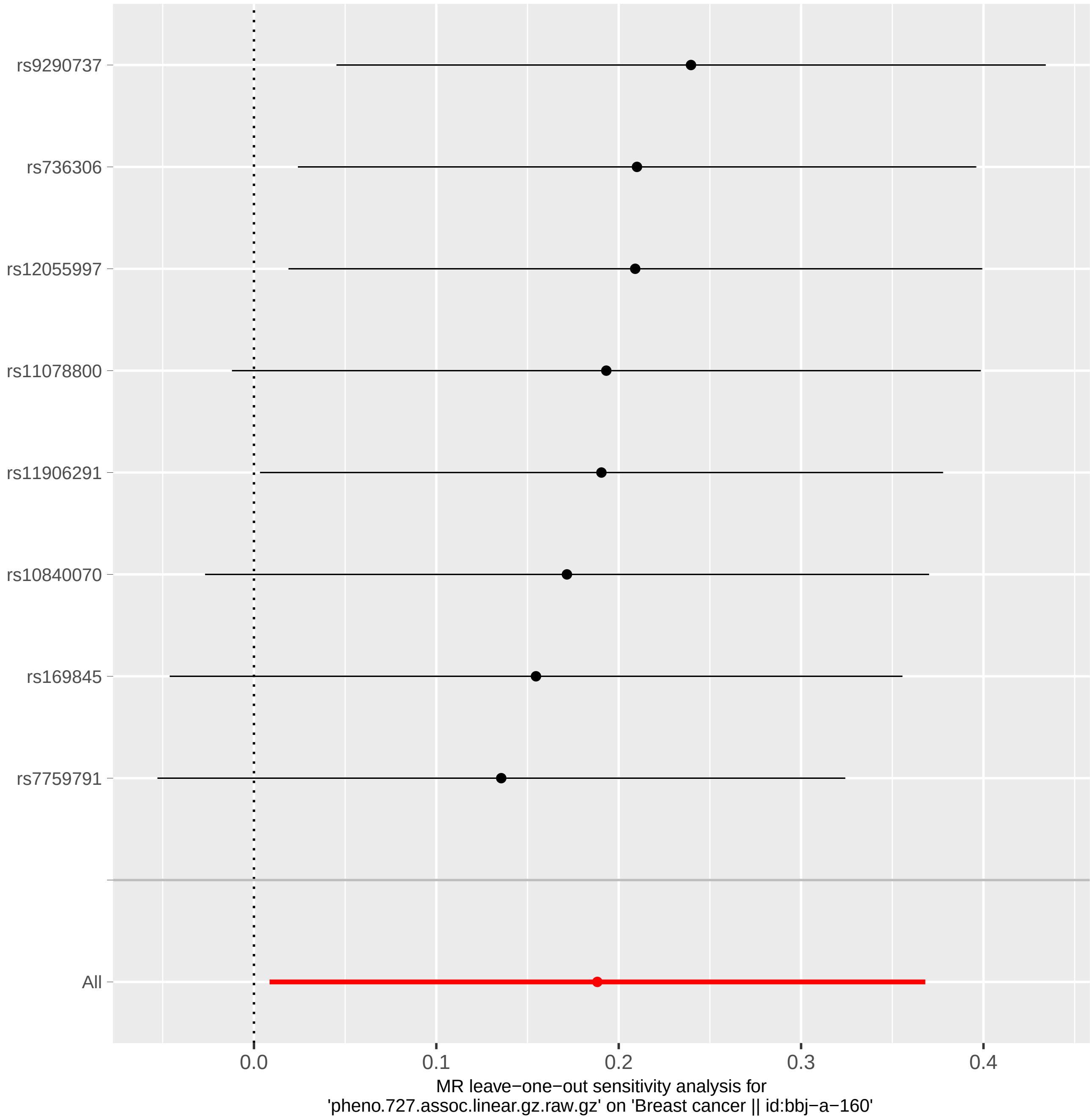

rs9290737
rs736306
rs12055997
rs11078800
rs11906291
rs10840070
rs169845
rs7759791
All
0.0
0.1
0.2
0.3
0.4
MR leave−one−out sensitivity analysis for
'pheno.727.assoc.linear.gz.raw.gz' on 'Breast cancer || id:bbj−a−160'
